# Vitamin D supplementation for children with cancer: A systematic review and consensus recommendations

**DOI:** 10.1002/cam4.4013

**Published:** 2021-06-08

**Authors:** Jenneke E. van Atteveld, Iris E. Verhagen, Marry M. van den Heuvel‐Eibrink, Hanneke M. van Santen, Inge M. van der Sluis, Natascia Di Iorgi, Jill H. Simmons, Leanne M. Ward, Sebastian J.C.M.M. Neggers

**Affiliations:** ^1^ Princess Máxima Center for Pediatric Oncology Utrecht The Netherlands; ^2^ Department of Endocrinology Wilhelmina Children's Hospital Utrecht The Netherlands; ^3^ Department of Pediatrics University of Genova IRCCS Istituto Giannina Gaslini Genova GE Italy; ^4^ Department of Pediatrics Vanderbilt University Medical Center Nashville TN USA; ^5^ Department of Pediatrics University of Ottawa Children's Hospital of Eastern Ontario Ottawa ON Canada; ^6^ Department of Endocrinology Erasmus Medical Center Rotterdam The Netherlands

**Keywords:** acute lymphoblastic leukemia, bone mineral density, childhood cancer, fractures, vitamin D

## Abstract

**Background:**

Prevalent vitamin D deficiency (VDD) and low bone mineral density (BMD) have led to vitamin D supplementation for children with cancer, regardless vitamin D status. However, it remains unsettled whether this enhances bone strength. We sought to address this issue by carrying out a systematic review of the literature.

**Methods:**

We conducted a literature search using PubMed, Embase, and Cochrane databases. Studies including children up to 5 years after cancer therapy were assessed for the association between 25‐hydroxyvitamin D (25OHD) levels and BMD *Z*‐scores or fractures, and the effect of vitamin D supplementation on BMD or fractures. Evidence quality was assessed using the GRADE methodology.

**Results:**

Nineteen studies (16 observational and 3 interventional, mainly involving children with hematologic malignancies) were included. One study which analyzed 25OHD as a threshold variable (≤10 ng/ml) found a significant association between 25OHD levels and BMD *Z*‐scores, while 25OHD as a continuous variable was not significantly associated with BMD *Z*‐scores in 14 observational studies. We found neither a significant association between lower 25OHD levels and fractures (2 studies), nor between vitamin D (and calcium) supplementation and BMD or fracture frequency (3 studies) (very low quality evidence).

**Conclusion:**

There is a lack of evidence for an effect of vitamin D (and calcium) supplementation on BMD or fractures in children with cancer. Further research is needed; until then, we recommend dietary vitamin D/calcium intake in keeping with standard national guidelines, and periodic 25OHD monitoring to detect levels <20 ng/ml. Vitamin D/calcium supplementation is recommended in children with low levels, to maintain levels ≥20 ng/ml year‐long.

## Graphical abstract

The relationship between 25OHD levels and BMD or fractures has been inadequately studied, and there is lack of evidence for an effect of universal vitamin D supplementation on BMD or fractures in children with cancer. Further research is needed; we provide recommendations to ensure 25OHD levels adequate for bone health in this population based on the current very low quality evidence and expert opinion.

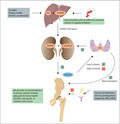

## 1 INTRODUCTION

Improved treatment strategies have substantially increased survival rates for childhood cancer over the past decades. The 5‐year survival rate is currently >80% and the majority of children are cured.^1^ However, this improved survival comes at a cost, as it is often accompanied by treatment‐related morbidity.^2^ One of these side effects is low bone mineral density (BMD). Low BMD may already be present at cancer diagnosis, for example due to the malignancy itself,^3^, ^4^ but is also common among survivors of childhood cancer due to cancer treatment or its consequences.^5^, ^6^, ^7^ Low BMD is associated with an increased risk of fractures in children with cancer^8^, ^9^ and in childhood cancer survivors.^10^ These fractures may lead to significant morbidity, hospitalization, and decreased quality of life.^11^

In the general pediatric population, BMD and fractures are influenced by multiple factors, such as sex, age, and weight.^12^ In addition, low BMD and fractures can partly be attributed to vitamin D deficiency (VDD).^13^, ^14^ Vitamin D (derived from ultraviolet radiation or dietary intake) is converted in the liver to 25‐hydroxyvitamin D (25OHD), and is further hydroxylated in the kidney to the active metabolite 1,25‐dihydroxyvitamin D (1,25[OH]_2_D). Low 25OHD levels decrease calcium and phosphate absorption and lead to an acute compensatory rise in parathyroid hormone (PTH), resulting in bone resorption to release calcium. Persistent VDD results in excessive bone resorption, generalized BMD decline, and bone mineralization defects. However, there remains some controversy around optimal and deficient serum 25OHD levels, mainly due to the large variability of 25OHD levels across commonly used assays and different races.^15^, ^16^, ^17^ Generally, serum 25OHD levels lower than 12 ng/ml (30 nmol/L) are associated with deficiency, but levels between 12 and 20 ng/ml (30–50 nmol/L) are already considered inadequate for bone strength in children.^14^, ^18^

Vitamin D deficiency occurs mainly due to decreased sunlight exposure, inadequate dietary intake, malabsorption, or liver and renal diseases.^19^ Children with cancer are therefore theoretically at risk for VDD, and some studies have shown that VDD is indeed more prevalent among children with hematologic malignancies compared to healthy children.^20^, ^21^, ^22^ The high prevalence of VDD and low BMD has led clinicians to often advise vitamin D supplements to children with cancer. In non‐cancer populations, vitamin D and calcium supplementation may increase BMD in children^23^ and in adults^24^ with low vitamin D levels, and can prevent fractures in adults.^19^, ^25^ In children with cancer, however, multiple disease‐ and treatment‐related risk factors for developing low BMD, such as cranial irradiation and glucocorticoids, have been described (in addition to the risk factors in the general population).^26^, ^27^ The relative contribution of these risk factors to low BMD, as well as their potential confounding effect on the association between VDD and BMD, are unclear. Therefore, it remains unsettled whether vitamin D supplementation in all children with cancer, regardless their vitamin D status, enhances bone strength. The aim of this systematic review was to assess the influence of VDD on the risk of low BMD and fractures, as well as the effect of vitamin D supplementation on BMD and fractures in children with cancer up to five years after the completion of therapy.

## 2 METHODS

This systematic review was prepared according to the Preferred Reporting Items for Systematic Reviews and Meta‐Analyses (PRISMA) statement.^28^

### 2.1 Search strategy and selection

We conducted a systematic literature search in PubMed, Embase, and Cochrane databases until August 2019. Search terms included children with cancer, survivors of childhood cancer, vitamin D serum concentration, and low BMD or fractures, and all related synonyms (Table S1). After removal of duplicates, the title and abstract of the retrieved records were screened to identify articles that would potentially match our predetermined inclusion criteria: (1) the study population consisted of children with cancer until five years after treatment cessation, with at least 95% of the population diagnosed at ≤18 years of age; (2) the study assessed the relationship between serum 25OHD levels and BMD *Z*‐scores (measured by dual‐energy X‐ray absorptiometry [DXA], quantitative computed tomography [QCT], or quantitative ultrasound [QUS]), or the relationship between 25OHD levels and fractures, or the effect of vitamin D supplementation (all forms) on BMD (raw value or *Z*‐score) change and/or fracture frequency; (3) the study did not exclusively or mainly report on BMD after hematopoietic stem cell transplantation; (4) the study was not a case report or case series (*n* <10) and was written in English; and (5) the study was original research. We only included studies measuring 25OHD (and not 1,25[OH]_2_D), as serum 25OHD levels are considered the best clinical indicator of vitamin D status (in patients with normal kidney function).^29^, ^30^ We excluded studies in childhood cancer survivors starting more than five years after treatment cessation because we aimed to assess the rationale and effect of vitamin D supplementation on bone health during cancer treatment. Before exclusion of reviews, the reference list was screened for relevant articles. Subsequently, full‐text articles were obtained and assessed according to the inclusion criteria. When multiple articles reported on the same cohort, we included the article that reported the most relevant data to our research questions. Finally, we performed a cross‐reference check on all included articles using Web of Science. Article screening was independently executed by two reviewers, JEvA and IEV, whereas disagreements were resolved by consensus or consultation of a third reviewer (SJCMMN).

### 2.2 Data extraction

We retrieved data on the sample size, sex distribution, age at baseline, country, study design, childhood cancer diagnosis, BMD imaging modality and skeletal site, and follow‐up duration from all included studies.

For observational studies, we additionally retrieved data on VDD threshold, the percentage of children receiving vitamin D supplementation, and the prescribed dose. As outcome measures, the difference between the percentage of children with low (areal and/or volumetric) BMD (aBMD and/or vBMD *Z*‐score ≤ −1 or ≤ −2) or fractures by vitamin D status (VDD yes vs. no), risk estimates for low BMD or fractures by vitamin D status, mean or median 25OHD levels, mean or median aBMD and vBMD *Z*‐scores at each timepoint, the percentage of children with any fracture in the whole study population, and the association between (change in) 25OHD levels and aBMD and vBMD *Z*‐scores and fractures were extracted if reported in the study.

For interventional studies, if available, we additionally retrieved data on supplementation, the percentage of children with low aBMD and/or vBMD per skeletal site or fractures, risk estimates for low BMD and fractures, and the mean difference of BMD values (g/cm^2^_,_ mg/cm^3^, or *Z*‐score) between baseline and follow‐up in the intervention and control group. Also, the *p*‐value of the effect of the intervention on BMD and fractures was extracted.

### 2.3 Critical appraisal

The same two independent reviewers (JvA and IEV) assessed the validity of the included articles with the Quality in Prognostic Studies (QUIPS) tool for observational studies and the Cochrane risk of bias tool for interventional studies.^31^, ^32^ The quality of the total body of evidence was assessed using the Grading of Recommendations Assessment, Development and Evaluation (GRADE) methodology.^33^ Discrepancies in the grading were resolved by consensus or consultation of a third reviewer (SJCMMN).

### 2.4 Consensus recommendations

Our panel consisted of experts in the field of pediatric oncology and endocrinology, in particular bone health and disease, representing four different countries and two different continents. Recommendations were drafted based on the evidence, expert opinion, as well as other considerations such as costs and applicability across different health‐care systems. Unanimous agreement was reached for all recommendations by a digital consensus meeting on October 13, 2020 in combination with rigorous pre‐ and post‐meeting revisions.

## 3 RESULTS

### 3.1 Search results

**FIGURE 1:**
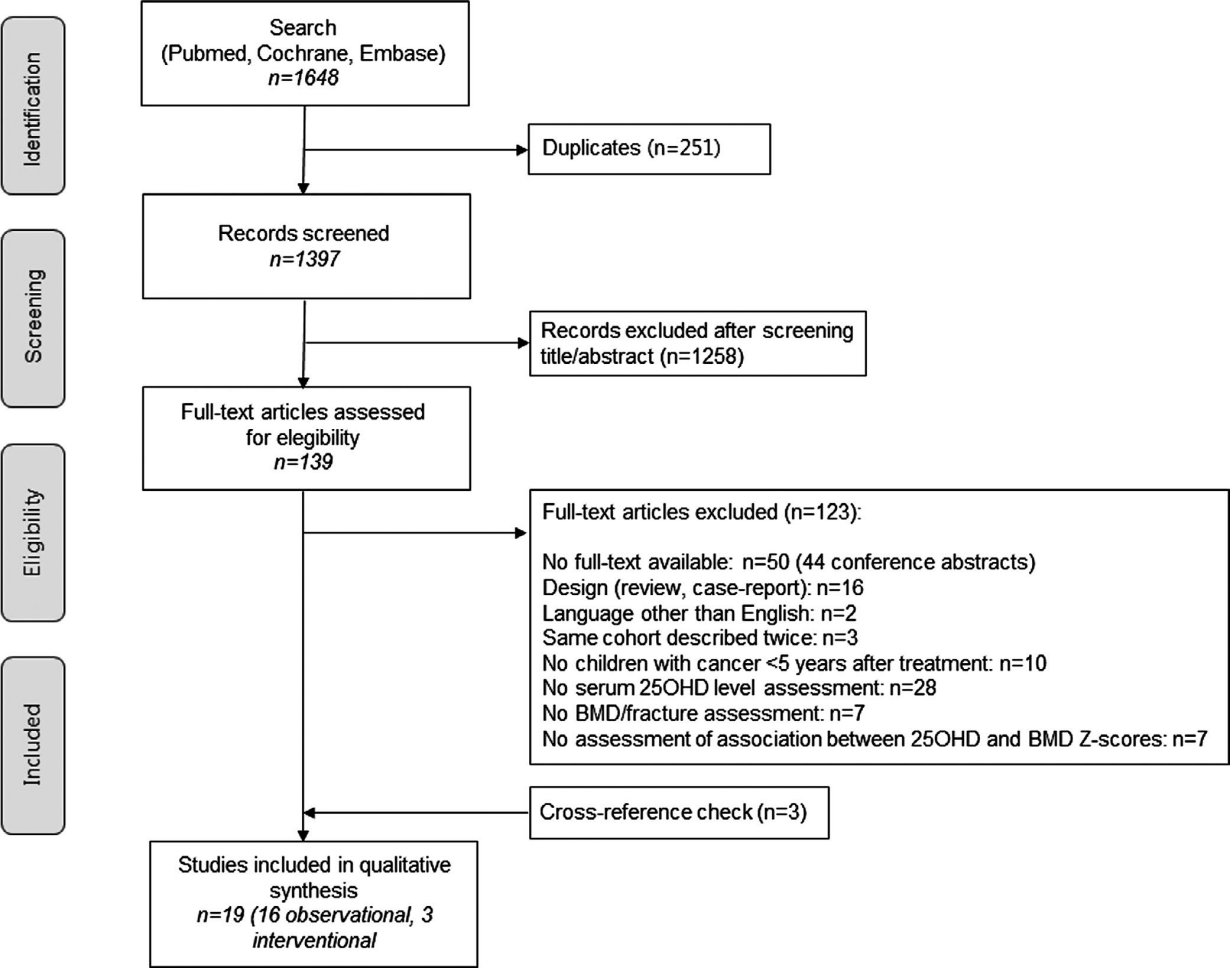
PRISMA flow diagram of study selection

The search in PubMed, Embase, and Cochrane yielded 320, 1219, and 109 records, respectively. After duplicate removal, 1397 titles and abstracts were screened and subsequently, 139 full‐text articles were reviewed (Figure 1). Sixteen articles were eligible for analysis; a cross‐reference check retrieved three additional articles. A total of 19 articles, including 16 observational studies^21^, ^34^, ^35^, ^36^, ^37^, ^38^, ^39^, ^40^, ^41^, ^42^, ^43^, ^44^, ^45^, ^46^, ^47^, ^48^, ^49^ and three interventional studies,^50^, ^51^, ^52^ were included in this review.

### 3.2 Study characteristics

Of the 16 observational studies, 11 studies^21^, ^36^, ^37^, ^38^, ^39^, ^40^, ^42^, ^43^, ^46^, ^48^, ^49^ (69%) were conducted in children with a hematologic malignancy, two studies^34^, ^41^ (13%) in children with solid tumors, and three studies^35^, ^45^, ^47^ (19%) in children with any childhood cancer diagnosis (Table 1). Nine studies^36^, ^37^, ^38^, ^40^, ^41^, ^43^, ^47^, ^48^, ^49^ (56%) had a cross‐sectional and seven studies^21^, ^34^, ^35^, ^39^, ^42^, ^45^, ^46^ (44%) a longitudinal design. Sample sizes of the studies varied considerably from 20 to 171 patients. Median or mean age at baseline of the study population ranged from 3.9 to 15.0 years. The serum 25OHD threshold for VDD was not consistent among the studies; 25OHD levels <20 ng/ml were most frequently used (55% of the studies that defined a threshold).^34^, ^40^, ^41^, ^43^, ^45^, ^47^ Four studies (36%) used a threshold of 12 ng/ml or lower.^36^, ^38^, ^42^, ^49^ aBMD *Z*‐scores of the lumbar spine (LS), total body (TB), total body less head (TBLH), and total hip (TH) and/or femoral neck (FN) were ascertained by DXA in 15 studies^21^, ^34^, ^35^, ^36^, ^37^, ^38^, ^39^, ^41^, ^42^, ^43^, ^49^ (94%) and vBMD *Z*‐scores of the femur by QCT in one study^40^ (6%). In addition, one study calculated height‐adjusted (i.e., apparent vBMD) *Z*‐scores.^36^ The frequency of symptomatic fractures (all types, diagnosed due to pain) was reported in six studies^34^, ^36^, ^37^, ^39^, ^40^, ^42^ (38%), of which two studies^34^, ^39^ (13%) assessed the association between serum 25OHD levels and fractures.

**TABLE 1:** Study characteristics of the observational studies in children during or shortly after cancer treatment

Author (year)	No. of patiënts	Sex (M)	Age at baseline (years)	Country^a^	Design	Childhood cancer diagnosis	25OHD VDD threshold^b^	Vit D suppl. (%, dose)	BMD modality and site; fractures	Timepoints (months)
*Hematologic malignancies*										
Boot 1999	32	21	Mean: 7.9	NL	L	ALL	<12 ng/ml	NR	Modality: DXA Site: LS, TB Fractures: +	Dx, 6, 12, 24, 12 after Rx
Bordbar 2016	60	39	Mean: 9.9	IR	C	ALL	<20 ng/ml	100% 200 IU/day	Modality: DXA Site: LS, FN Fractures: NR	6 after Dx
El‐Ziny 2005	43	23	Mean: 7.0	EG	L	Acute leukemia	NR	NR	Modality: DXA Site: LS Fractures: NR	Dx, 3, 12
El‐Ziny 2007	20	11	Mean: 8.9	EG	L	Malignant lymphoma	NR	NR	Modality: DXA Site: LS Fractures: NR	Dx, 3, 12
Gunes 2010	70	41	Mean: 10.6	TR	C	ALL	NR	NR	Modality: DXA Site: LS Fractures: NR	45.5 after Rx
Halton 1995	40	27	Median: 3.9	CA	C	ALL	<10 ng/ml	NR	Modality: DXA Site: LS Fractures: NR	Dx
Jain 2017	65	52	Median: 15.0	IN	C	ALL	<10 ng/ml	NR	Modality: DXA Site: LS, TB Fractures: +	52 after Rx
Kadan‐Lottick 2001	75	NA	Mean: 6.8	US	C	ALL	NR	NR	Modality: DXA Site: TB Fractures: +	30 after Rx
Kelly 2009	41	25	Median: 10	CO	C	ALL	<9 ng/ml	NR	Modality: DXA Site: TB Fractures: NR	During or after completion of Rx
Marinovic 2005	37	20	Median: 7.9	FR	L	ALL	NR	NR	Modality: DXA Site: LS, TB Fractures: +	26 after Rx, +12
Mostoufi‐Moab 2012	50	19	Median: 7.9	US	C	ALL	<20 ng/ml	40% 400 IU/day	Modality: QCT Site: tibia Fractures: +	10 after Rx, +12
*Solid tumors*										
Bilariki 2010	52	30	Median: 12.1	FR	L	Solid tumor	<20 ng/ml	80% 100,000 IU/3 months	Modality: DXA Site: LS, TH Fractures: +	13.8 after Rx, +12
Saki 2018	50	36	Mean: 10.3	IR	C	Solid tumor	<20 ng/ml	100% 200 IU/day	Modality: DXA Site: LS, FN, TH Fractures: NR	During or after completion of Rx
*Any childhood cancer diagnosis*										
Choi 2017	30	21	Median: 11.2	KR	L	Any type	<20 ng/ml	NR	Modality: DXA Site: TB Fractures: NR	Dx, 1, 6, 12
Esbenshade 2014	171	96	Median: 12.1	US	C	Any type	<20 ng/ml	1.2% Dose NR	Modality: DXA Site: LS, TB Fractures: NR	2.7 after Rx
Henderson 1998	37	NA	Mean: 7.3	US	L	Any type	<15 ng/ml	NR	Modality: DXA Site: LS, TH Fractures: NR	Dx, 5—8 interval

Abbreviations: ALL, acute lymphoblastic leukemia; BMD, bone mineral density; C, cross‐sectional; Dx, diagnosis; DXA, dual‐energy X‐ray absorptiometry; FN, femoral neck; IU, international units; L, longitudinal; LS, lumbar spine; M, male; NR, not reported; QCT, quantitative computed tomography; Rx, treatment; TB, total body; TBLH, total body less head; TH, total hip.

^a^ International Organization of Standardization (ISO) country codes.

^b^ 1 ng/ml = 2.5 nmol/L.

All three interventional studies (two open‐label RCTs^50^, ^52^ and one quasi‐experimental study^51^) were performed in children with acute lymphoblastic leukemia (ALL; Table 2). Sample sizes ranged from 16 to 115 children. Age at start of the intervention ranged from 3.7 to 15.2 years and the duration of the intervention ranged from 6.7 to 12 months. Vitamin D was supplemented in combination with calcium during the first phases of ALL treatment in all children in two studies^50^, ^51^ (67%) and in children with 25OHD levels <30 ng/ml in one study^52^ (33%). The formulation (vitamin D3 vs. the active form of vitamin D, 1,25[OH]_2_D) and vitamin D supplement doses (400–600 IU/day vs. 10,000 IU every 2 months oral vitamin D3 vs. 10–20 IU/day 1,25[OH]_2_D) varied. aBMD (g/cm^2^ or *Z*‐score) of the LS, TB, TBLH, and/or TH was measured by DXA in two studies^50^, ^51^ (67%), and vBMD (mg/cm^3^) of the LS and femur was measured by QCT in one study^52^ (33%). All three studies compared the frequency of symptomatic fractures in the intervention and control group.

**TABLE 2:** Study characteristics of the interventional studies in children during or shortly after cancer treatment

Author (year)	No. of participants	Sex (M)	Age at baseline (years)	Country^a^	Design	Childhood cancer diagnosis	Intervention group	Control group	Outcome	Follow‐up
*Hematologic malignancies*										
Demirsoy 2017	Intervention: 34	16	Median: 3.7	TR	Quasi‐experimental study	ALL	Oral vitamin D3 (400–600 IU/day) + Ca carbonate (500–1,000 mg/day) supplementation	Historical controls without vitamin D/Ca supplementation	BMD (g/cm^2^, *Z*‐score) Modality: DXA Site: LS, TB, TBLH Fractures: +	From diagnosis until completion of reinduction therapy (~8 months)
	Controls: 59	34	Median: 8.9							
Díaz 2008	Intervention: 8	5	Mean total: 5.5	CL	RCT	ALL	Oral 1,25(OH)_2_D (10–20 IU/day) + Ca carbonate (500 mg/day) supplementation	Ca carbonate (500 mg/day) supplementation	BMD (g/cm^2^) Modality: DXA Site: LS, TB, TH Fractures: +	From diagnosis until 1 year into treatment
	Controls: 8	4								
Orgel 2017	Intervention: 19	13	Median: 15.2	US	RCT	ALL	Directly observed therapy: oral vitamin D3 (100,000 IU/2 months) + Ca carbonate (800 mg/day) in addition to standard of care	Standard of care: routine encouragement regarding activity and ad hoc nutritional monitoring	BMD (mg/cm^3^) Modality: QCT Site: LS, femur Fractures: +	From end of induction until delayed intensification (median 6.7 months)
	Controls: 10	6	Median: 14.6							

Abbreviations: ALL, acute lymphoblastic leukemia; BMD, bone mineral density; Ca, calcium; DXA, dual‐energy X‐ray absorptiometry; IU, international units; LS, lumbar spine; M, male; QCT, quantitative computed tomography; RCT, randomized controlled trial; TB, total body; TBLH, total body less head; TH, total hip.

^a^ International Organization of Standardization (ISO) country codes.

### 3.3 Study quality

There were significant concerns about the risk of bias in the included studies (Tables S2 and S3). The main limitations of the observational studies concerned low study participation rates, inadequate prognostic factor measurement (25OHD not measured by liquid chromatography‐tandem mass spectrometry [gold standard] and/or analyzed at different timepoints), lack of adjustment for important confounders (no multivariable analysis), and suboptimal statistical analysis or reporting (correlations instead of risk estimates using a 25OHD and BMD *Z*‐score threshold). The main limitations of the interventional studies concerned a lack of adequate randomization procedures, allocation concealment, or blinding of participants and personnel, as well as incomplete outcome data.

### 3.4 Vitamin D status, BMD status, and fractures

Mean or median 25OHD levels and BMD *Z*‐scores per timepoint are shown in Table 3. Mean or median 25OHD levels were below 20 ng/ml at one or more timepoints in seven studies^21^, ^34^, ^36^, ^39^, ^45^, ^46^, ^49^ (44%), and below 12 ng/ml in four studies^21^, ^39^, ^45^, ^46^ (25%). Mean or median aBMD *Z*‐scores at any skeletal site and at one or more timepoints were <0 in 12^21^, ^34^, ^35^, ^36^, ^38^, ^41^, ^42^, ^43^, ^45^, ^46^, ^48^, ^49^ of the 14 studies^21^, ^34^, ^35^, ^36^, ^37^, ^38^, ^41^, ^42^, ^43^, ^45^, ^46^, ^47^, ^48^, ^49^ (86%) that reported aBMD *Z*‐scores during or (just) after treatment. Two studies which reported apparent and true vBMD *Z*‐scores, respectively, found mean values below zero as well.^36^, ^40^ Because the timepoints as well as the 25OHD level threshold for VDD and BMD *Z*‐score threshold for low BMD varied across the studies and did not allow comparisons, no comprehensive overview of the percentage of children with VDD or low BMD in the included studies was calculated. In addition, none of the included studies compared the incidence of symptomatic fractures with a healthy reference population, so we could not determine the incidence rate ratio of fractures in children with cancer.

**TABLE 3:** Results of the observational studies

	25OHD levels (mean [SD]) in ng/ml or nmol/L*	LS aBMD *Z*‐scores (mean [SD])	TB or TBLH* aBMD *Z*‐scores (mean [SD])	TH aBMD *Z*‐scores (mean [SD])	FN aBMD *Z*‐scores (mean [SD])	Fractures (%)	Association
*Hematologic malignancies*							
Boot 1999						16%	No significant correlation^a^
T1	115 (67)*	−0.67 (1.3)	0.02 (1.3)	NR	NR		
T2	60 (26)*	NR	NR	NR	NR		
T3	79 (36)*	NR	NR	NR	NR		
T4	63 (30)*	NR	NR	NR	NR		
T5	56 (32)*	NR	NR	NR	NR		
Bordbar 2016						NR	No significant association^b^
T1	20.4 (15.2)	−1.3 (1.2)	NR	NR	−1.9 (1.3)		
El‐Ziny 2005						NR	No significant correlation^c^
T1	11.0 (5.3 to 29.0)^d^	−1.8 (−3.0 to −0.1)^d^	NR	NR	NR		
T2	14.2 (5.5 to 26.8)^d^	−1.1 (−2.0 to −0.4)^d^	NR	NR	NR		
T3	17.5 (10.3 to 38.5)^d^	−1.1 (−1.9 to −0.4)^d^	NR	NR	NR		
El‐Ziny 2007						NR	No significant correlation^c^
T1	8.5 (6.7 to 21.0)^d^	−0.3 (−1.6 to 0.6)^d^	NR	NR	NR		
T2	29.0 (16.0 to 49.0)^d^	−0.7 (−2.3 to 0.6)^d^	NR	NR	NR		
T3	12.0 (10.0 to 29.0)^d^	−0.9 (−2.2 to 0.6)^d^	NR	NR	NR		
Gunes 2010						NR	No significant correlation (*p* = 0.06)^c^ and association (*p*‐value NR)
T1	21.0 (7.9)	−1.72 (0.83)	NR	NR	NR		
Halton 1995					`	10%	No significant correlation^c^
Total	17.0 (15.2)	NR	NR	NR	NR		
Boys	NR	−0.16	NR	NR	NR		
Girls	NR	−0.76	NR	NR	NR		
Jain 2017						18%	No significant association: *p* = 0.196 (LS) *p* = 0.068 (LS HA) *p* = 0.089 (TB)^e^ Significant association: *p* = 0.046 (TB HA)^e^
T1	29.5 (35.9)*	−1.24 (1.21) −0.67 (1.11)^f^	−0.91 (1.00) −0.84 (0.92)^f^	NR	NR		
Kadan‐Lottick 2001						28%	No significant association: *p* = 0.2^g^
Total	NR	NR	0.22 (0.96)	NR	NR		
BMD *Z*‐score ≤ −1	43 (17)	NA	NA	NA	NA		
BMD *Z*‐score > −1	37 (11)	NA	NA	NA	NA		
Kelly 2009						NR	No significant correlation^c^
Total	23.1 (6.0 to 36.9)	NR	NR	NR	NR		
On Rx <12 months (17%)	NR	NR	−0.46 (0.48)	NR	NR		
On Rx >12 months (41%)	NR	NR	−1.72 (0.33)	NR	NR		
Off Rx >12 months (41%)	NR	NR	−0.41 (0.31)	NR	NR		
Marinovic 2005						22%	No significant association^h^
Total	NR	NR	NR	NR	NR		
Fracture + (22%)	10 (8.5 to 16.5)^d^	NA	NA	NA	NA		
Fracture – (78%)	10.5 (8 to 16)^d^	NA	NA	NA	NA		
Mostoufi‐Moab 2012						18%	No significant association^c^
T1	30.9 (4.1 to 93.6)	NR	−0.84 (1.05)^i^	NR	NR		
T2	NR	NR	−0.51 (0.91)^i^	NR	NR		
*Solid tumors*							
Bilariki 2010						21%	No significant correlation^j^ Significant association: *p* = 0.002^k^
T1	19.7 (8.5)	−0.86 (1.11)	NR	−0.87 (0.98)	NR		
T2	20.5 (7.1)	NR	NR	NR	NR		
Fracture + (21%)	23.7 (7.4)	NA	NA	NA	NA		
Fracture – (79%)	18.7 (8.4)	NA	NA	NA	NA		
Saki 2018						NR	No significant association: *p* = 0.991 *p* = 0.717^l^
T1	23.3 (18.3)	−1.4 (1.4)	NR	−1.6 (0.9)	−1.8 (1.3)		
*Any childhood cancer diagnosis*							
Choi 2017						NR	No significant association^m^
Hematologic T1	12.6 (4.4 to 22.2)^d^	NR	0.70 (−1.40 to 2.50)^d^	NR	NR		
Hematologic T2	NR	NR	0.65 (−1.5 to 2.5)^d^	NR	NR		
Hematologic T3	NR	NR	0.10 (−1.6 to 1.3)^d^	NR	NR		
Hematologic T4	NR	NR	−0.80 (−1.7 to 1.3)^d^	NR	NR		
Solid T1	11.9 (9.3 to 47.9)^d^	NR	0.00 (−1.4 to 1.8)^d^	NR	NR		
Solid T2	NR	NR	−0.20 (−1.1 to 1.9)^d^	NR	NR		
Solid T3	NR	NR	−0.60 (−1.9 to 1.8)^d^	NR	NR		
Solid T4	NR	NR	−0.70 (−2.1 to 1.8)^d^	NR	NR		
Esbenshade 2014						NR	No significant correlation: ρ = 0.10, *p* = 0.374 (TB) ρ = 0.09, *p* = 0.39 (LS)^c^ No significant association: *p* = 0.32^g^; *p* = 0.41^n^ (TB) *p* = 0.81^g^; *p* = 0.16^n^ (LS)
T1	29 (6 to 82)^d^	0.0 (−4.2 to 3.3)^d^	0.1(−4.2 to 3.6)^d^	NR	NR		
Henderson 1998						NR	No significant correlation^o^
T1	NR	−0.46 (0.22)^p^	NR	−0.60 (0.21)^p^	NR		
T2	NR	NR	NR	NR	NR		
T3	NR	NR	NR	NR	NR		
T5	NR	−0.37 (0.27)^p^	NR	−0.48 (0.24)^p^	NR		

Abbreviations: aBMD, areal bone mineral density; HA, height‐adjusted; LS, lumbar spine; NA, not applicable; NR, not reported; Rx, treatment; SD, standard deviation; SE, standard error; T, time‐point; TB, total body; TBLH, total body less head.

^a^ Between 25OHD levels and BMD *Z*‐scores at diagnosis, during, and after treatment.

^b^ Between 25OHD levels and LS and FN BMD *Z*‐scores.

^c^ Between 25OHD levels and BMD *Z*‐scores.

^d^ Median (range).

^e^ Between low vitamin D levels (≤25 nmol/L) and BMD *Z*‐scores.

^f^ Height‐adjusted BMD *Z*‐score.

^g^ Between 25OHD levels and BMD *Z*‐score ≤ ‐1.

^h^ Between 25OHD levels in patients with and without fractures.

^i^ Tibial cortical vBMD *Z*‐score.

^j^ Between Δ25OHD levels and ΔBMD *Z*‐scores.

^k^ Significantly higher 25OHD levels in patients with fractures.

^l^ Between 25OHD levels and LS and FN BMD *Z*‐score ≤ ‐2.

^m^ Between 25OHD and BMD *Z*‐score at diagnosis.

^n^ Between 25OHD levels and BMD *Z*‐score ≤ ‐2.

^o^ Between 25OHD levels and ΔBMD *Z*‐scores.

^p^ Mean (SE).

### 3.5 Association between 25OHD levels and BMD *Z*‐scores

None of the included studies assessed the association between VDD (using the threshold defined in the study) and low BMD (using a *Z*‐score threshold) or fractures. Therefore, it was not possible to provide risk estimates for low BMD and fractures in children with VDD. In a study of 65 childhood ALL survivors, Jain et al^36^ reported a significant association (*p* = 0.046) between low 25OHD levels (≤10 ng/ml, *n* = 36) and lower height‐adjusted TB BMD *Z*‐scores (continuous) at a median of 52 months after cessation of treatment. However, there was no significant association between low 25OHD levels and height‐adjusted LS, non‐height adjusted LS, or TB BMD *Z*‐scores. All 14 studies^21^, ^34^, ^35^, ^37^, ^38^, ^40^, ^41^, ^42^, ^43^, ^45^, ^46^, ^47^, ^48^, ^49^ that assessed the association between 25OHD levels as a continuous variable and BMD *Z*‐scores found no significant association (Table 3).

According to the GRADE assessment, there is very low quality evidence with conflicting results for the association between lower 25OHD levels and lower BMD *Z*‐scores in children with cancer up to five years after cancer treatment (Table S4).

### 3.6 Association between 25OHD levels and fractures

Two studies^34^, ^39^ assessed the association between vitamin D levels and symptomatic fractures (Table 3). Marinovic et al^39^ did not find a significant association between mean 25OHD levels in 37 children with ALL with (22%) and without (78%) a history of symptomatic fractures in the previous five years (10.0 vs. 10.5 ng/ml) from diagnosis until a median follow‐up of 38 months after cessation of treatment. Bilariki et al^34^ reported significantly higher mean levels of 25OHD at 13.8 months after treatment in 10 out of 52 children with a solid tumor who experienced symptomatic fractures from diagnosis until follow‐up compared to those without fractures (23.7 vs. 18.7 ng/ml, *p* = 0.002).

According to the GRADE assessment, very low quality evidence suggests that there is no increased risk of fractures for children with lower 25OHD levels up to five years after cancer treatment (Table S4).

### 3.7 Effect of vitamin D supplementation on BMD and fractures

Table 4 summarizes the results of the three interventional studies in children with ALL. Demirsoy et al^51^ reported a significant increase in median (interquartile range, IQR) 25OHD levels in the intervention group from ALL diagnosis until completion of reinduction therapy (17.9 [IQR 10.9 to 23.7] vs. 23.5 [IQR 19.9 to 28.6] ng/ml, *p* = 0.01). However, median BMD *Z*‐score decreased significantly during this interval (LS BMD *Z*‐score −0.6 [IQR −1.1 to 0.2] vs. −1.6 [IQR −2.1 to −0.1], *p* = 0.025; TB BMD *Z*‐score 0.1 [IQR −0.5 to 0.9] vs. −0.7 [IQR −1.4 to 0.1], *p* = 0.005; TBLH BMD *Z*‐score 0.2 [IQR −0.2 to 1.5] vs. −0.5 [IQR −1.7 to 0.0], *p* = 0.005). The study design did not allow a comparison of the difference of 25OHD levels and BMD during supplementation with the control group. Diaz et al^50^ and Orgel et al^52^ both found a greater increase or smaller decrease in BMD during the study period in the control group compared to the intervention group, indicating that the intervention was not effective. In all three studies, the percentage of children with symptomatic fractures was equal or higher in the interventional group compared to the control group.^50^, ^51^, ^52^

**TABLE 4:** Results from the interventional studies

		Intervention group (mean ± SD)			Control group (mean ± SD)			*p*‐value
		Baseline	End of study	Δ	Baseline	End of study	Δ	
*Hematologic malignancies*								
Demirsoy 2017	LS BMD^a^ TB BMD^a^ TBLH BMD^a^ Fractures^b^	−0.6 0.1 0.2 NA	−1.6 −0.7 −0.5 NA	−1.0 −0.8 −0.7 6%	NR NR NR NA	NR NR NR NA	NR NR NR 2%	NR NR NR NR
Díaz 2008	LS BMD^c^ TB BMD^c^ TH BMD^c^ Fractures^b^	NR NR NR NA	NR NR NR NA	83 −73 16 0%	NR NR NR NA	NR NR NR NA	101 26 31 0%	0.637 0.834 0.834 NR
Orgel 2017	LS vBMD^d^ Femoral vBMD^d^ Fractures^b^	249.3 ± 71.0 2091.4 ± 43.5 NA	203.8 ± 77.1 2093.1 ± 62.5 NA	−45.5 1.7 0%	234.6 ± 52.0 2081.7 ± 66.2 NA	201.4 ± 66.4 2090.9 ± 26.7 NA	−33.2 9.2 0%	0.432 0.915 NR

Abbreviations: BMD, bone mineral density; LS, lumbar spine; NA, not applicable; NR, not reported; TB, total body; TBLH, total body less head; SD, standard deviation; vBMD, volumetric bone mineral density.

^a^ BMD *Z*‐score.

^b^ Symptomatic fractures (pain).

^c^ BMD in g/cm^2^.

^d^ vBMD in mg/cm^3^.

According to the GRADE assessment, very low quality evidence suggests that there is no significant effect of vitamin D supplementation on BMD and fracture frequency in children with ALL up to five years after cancer treatment compared to controls (Table S4).

### 3.8 Consensus recommendations

Table 5 shows our consensus recommendations to ensure an adequate vitamin D status in the context of bone health in children with cancer, which are mainly based on expert opinion (supported by international guidelines for the general population) as a result of the very low quality evidence identified by this systematic review. In summary, we recommend to encourage a diet adequate in calcium and vitamin D according to standard national guidelines (expert opinion), and to monitor 25OHD levels at diagnosis with subsequent measurements every 6 months at least throughout therapy (expert opinion). Vitamin D ± calcium supplementation is recommended in children with 25OHD levels <20 ng/ml (very low quality evidence and expert opinion).

**TABLE 5:** Consensus recommendations to ensure an adequate vitamin D status in the context of bone health in children with cancer

We recommend adequate dietary vitamin D and calcium, i.e., 400 IU vitamin D and 200–1,000 mg calcium (depending on age) per day, as recommended by the IOM. In addition, if national guidelines on vitamin D supplementation for certain groups (e.g., infants) in the general population are present, these also apply to children with cancer (expert opinion, supported by the IOM 2011 guideline^18^)
We recommend to monitor 25OHD at cancer diagnosis with subsequent measurements every 6 months, at least until cessation of treatment, in all children with cancer (expert opinion)
We recommend (additional) vitamin D (D2 or D3) supplementation in children with 25OHD levels below 20 ng/ml (initial dose: 2,000 IU/day) throughout treatment, or higher doses if serum levels >20 ng/ml are not reached after 3 months (very low quality evidence and expert opinion). In addition, if the recommended daily amount of dietary calcium is not met, we recommend 500 mg calcium supplementation per day (expert opinion)

Abbreviations: IOM, institute of medicine; IU, international units; 25OHD, 25‐hydroxyvitamin D.

## 4 DISCUSSION

In adult childhood cancer survivors, there is a greater than expected proportion with BMD *Z*‐scores ≤ −1, and 10%–20% have BMD *Z*‐scores ≤ −2.^5^ The BMD trajectory in individual patients from cancer diagnosis until adulthood is still largely unknown. However, prevention of low BMD during therapy could conceivably reduce fracture risk in children with cancer and survivors. Patient‐specific risk factors (age, race, and sex, for example),^5^, ^11^ are non‐modifiable, and treatment‐specific risk factors are challenging to modify without adversely affecting remission and cure rates. However, vitamin D supplementation, if effective, would be a simple and inexpensive intervention. Based upon very low quality evidence overall, we identified inconsistent findings regarding the association between lower 25OHD levels and lower BMD *Z*‐scores, no significant association between lower 25OHD levels and fractures, and no significant effect of vitamin D supplementation on BMD and fractures in children with cancer (mainly hematologic malignancies) up to five years after cancer therapy. The very low quality of evidence calls into question whether the identified lack of effect is due to lack of evidence, or whether other factors explain the BMD decline and fractures in children with cancer, which effects are not modifiable by vitamin D supplementation.

The observational studies included in this review used different thresholds to define VDD. Fourteen studies assessed the association between 25OHD levels as a continuous variable and BMD *Z*‐scores and reported no significant association. Notably, the only study that assessed the association between VDD according to a threshold, in this case 25OHD levels ≤10 ng/ml, and BMD *Z*‐scores reported a significant association.^36^ It is important to note that using vitamin D as a continuum makes a meaningful evaluation of a potential association with BMD difficult. Although this methodology eliminates the problem of having to choose an arbitrary threshold for VDD, it is associated with another methodological issue: in the general population, a relationship between 25OHD and BMD has been observed in patients with vitamin D insufficiency or deficiency, but not in patients with a vitamin D replete state.^53^ Because most of the observational studies in this systematic review analyzed a correlation between 25OHD levels (including replete 25OHD values) and BMD *Z*‐scores, this might have led to false negative results.

Only two studies assessed the association between 25OHD levels and fractures. One study^34^ reported significantly higher mean levels of 25OHD in children with fractures compared to those without fractures. However, both studies measured 25OHD levels in the patients after the fractures (if present) had already occurred. This significant finding may thus reflect the fact that after the fracture had been diagnosed, vitamin D supplementation may have been more frequently recommended (and taken) in children with fractures compared to those without.

There was very low quality evidence to suggest that vitamin D supplementation has no significant effect upon BMD and fracture risk in children with ALL. These results are similar to those of an RCT in 275 long‐term childhood ALL survivors by Kaste et al., who found no significant effect of nutritional counseling with supplementation (1,000 mg/day calcium and 800 IU/day cholecalciferol) or placebo for two years on LS BMD *Z*‐scores.^54^ However, the doses of vitamin D supplementation utilized in the three included interventional studies varied significantly. Furthermore, most included studies were hampered by (very) small sample sizes, had a short follow‐up, were performed in children with leukemia and not with other types of cancer, and failed to adjust for important confounders such as body mass index (BMI) and skin tone. These limitations also apply to the observational studies.

In children and adults without cancer, large studies have established the relationship between VDD and bone mineralization defects (rickets and osteomalacia in children, osteomalacia in adults), generalized decrease in BMD, as well as muscle weakness, at a critical cut‐off of 12 ng/ml.^18^, ^19^ Recent meta‐analyses of vitamin D trials demonstrated that the effect of vitamin D supplementation on BMD and fracture risk is only significant in adults with baseline 25OHD levels lower than 16 ng/ml,^55^, ^56^ and a meta‐analysis in children identified a similar threshold.^23^ This indicates that there seems to be a minimum requirement of 25OHD, and that supplementation only benefits estimates of bone strength when this requirement is not met (i.e., in vitamin D deficient children). More recent studies also failed to show an effect of (high dose) vitamin D supplementation when applied to children generally (i.e., regardless their 25OHD status).^57^, ^58^

It is likely that low BMD and increased fracture risk in pediatric cancer patients and recent childhood cancer survivors is even more multifactorial in etiology than in the general population. The cancer itself, its treatment, or their consequences such as weakness of bone due to previous bone marrow infiltration by the oncologic disease, glucocorticoid use, osteotoxic effects of chemotherapy and radiotherapy, immobility, malnutrition, or endocrine deficiencies could be such additional (potentially confounding) etiologies.^5^, ^9^, ^26^, ^59^, ^60^, ^61^ These factors may impact BMD more severely and in a larger proportion of children with cancer than low vitamin D levels, and their effects on BMD and fracture risk may not be prevented or overcome by vitamin D supplementation alone.

This systematic review with consensus recommendations may be a first step towards the development of an evidence‐based clinical practice guideline for bone health in children with cancer. The knowledge gap that this systematic review has identified, could be overcome by prospective, adequately powered studies addressing the risk of low BMD (*Z*‐score ≤ −1 or ≤ −2) and fractures for children with cancer at different 25OHD cut‐offs, and the effect of vitamin D (and calcium) supplementation on estimates of bone strength. To provide guidance to clinicians until this new evidence has emerged, we have provided strong recommendations on the basis of the current very low quality evidence and expert opinion (supported by international guidelines for the general population).

We propose that ensuring adequate vitamin D status and mitigating modifiable bone problems in children with cancer are important. According to the Institute of Medicine (IOM), the minimal daily requirement of vitamin D and calcium in children is 400 IU and 200–1,100 mg (depending on age), respectively.^18^ A diet adequate in vitamin D and calcium should be encouraged.^18^, ^62^ Another natural way to acquire vitamin D is through sunlight exposure; however, we abstain from recommendations in this regard given the potential adverse effects on skin health.^63^ If national guidelines on vitamin D supplementation for certain groups (e.g., infants) in the general population are present, these also apply to children with cancer. For several reasons, it is conceivable that not all children with cancer will be able to meet the minimal daily requirement of vitamin D and calcium, at least not during all treatment phases. We suggest that in these children, it is reasonable to monitor the 25OHD status regularly instead of supplementing all children (although the harms and costs of standard supplementation appear minimal^23^), since the added benefit of vitamin D supplementation in children and adults with normal vitamin D levels has not been demonstrated,^23^, ^56^ and children with cancer undergo frequent phlebotomy. We therefore recommend measurement of 25OHD levels at cancer diagnosis with subsequent measurements every 6 months, at least until cessation of treatment. In addition, we think it is reasonable to continue 25OHD surveillance throughout the first years of follow‐up, however, the frequency may be lower as it may depend upon the frequency of follow‐up visits. Although elevated PTH (and alkaline phosphatase) levels provide definitive evidence of clinically significant VDD, we do not recommend universal PTH surveillance, amongst others due to financial constraints in some regions. However, measurement of PTH may be of additional value in children in whom VDD is clinically suspected or in situations when vitamin D concentrations may be unreliable, such as in children with obesity. In these cases, an elevated PTH level is helpful to diagnose VDD, and may diagnose VDD earlier, preventing more severe consequences.

In children with 25OHD levels below 20 ng/ml, we recommend supplementation with vitamin D (D2 or D3) throughout treatment at an initial dose of 2,000 IU vitamin D per day, as well as 500 mg calcium per day if the recommended daily amount of dietary calcium is not met. This is consistent with the widely‐used, global consensus statement in children without cancer by Munns et al.^14^ Measurement of 25OHD levels after 3 months could verify adequate dosing and compliance in patients receiving supplementation. Higher doses may be needed if serum 25OHD levels >20 ng/ml are not reached at this point. Each 1,000 IU/day of vitamin D3 in addition to what a child is currently ingesting will raise the level of 25OHD by 10 ng/ml after a few weeks.^64^ The BMI of the patient and the assay that was used need to be taken into consideration in this regard.^65^, ^66^ The risk of vitamin D toxicity is considered negligible using our recommended doses.^14^ A more extensive report on vitamin D monitoring, titration and its caveats, possible other beneficial effects of vitamin D than bone strength, as well as long‐term follow‐up recommendations,^67^ were neither within the scope of this systematic review nor our consensus recommendations.

In conclusion, this systematic review identified that the risk of low BMD during and shortly after cancer treatment for children with VDD has not yet been adequately studied. Very low quality evidence showed inconsistent results for the association between low vitamin D status and reductions in BMD parameters. Similarly, the relationship between 25OHD status and fractures as well as the effect of vitamin D supplementation has not been sufficiently studied to draw meaningful conclusions. Adequately powered prospective studies assessing the risk of low BMD and fractures for children with all types of cancer at different 25OHD cut‐offs, as well as the effect of vitamin D (and calcium) supplementation to improve the BMD–fracture pathway in this population are needed. On the other hand, it is well‐established that a small, critical amount of vitamin D is needed to prevent overt disturbances in mineral ion metabolism (i.e., hyperparathyroidism and hypocalcemia) in both the healthy and cancer setting. To prevent severe VDD causing overt skeletal effects, children should receive adequate intakes of calcium and vitamin D through diet to meet targets recommended by the IOM 2011 guidelines.^18^ Because of the frequency of VDD and low BMD in children on, or who have received, cancer therapy, children undergoing cancer therapy and recent childhood cancer survivors should have routine 25OHD surveillance in order to detect critical VDD that would require supplementation beyond routine preventative measures.

## CONFLICT OF INTEREST

All authors have nothing to disclose.

## ETHICAL STATEMENT

Ethical approval was not sought for this study because of its design (systematic review).

## Supporting information

Supplementary Material

## Data Availability

Not applicable (systematic review).
