# Supplementary material for: Vitamin D supplementation for children with cancer: A systematic review and consensus recommendations
Source: Cancer Med. 2021 Jun 8;10(13):4177–94. doi: 10.1002/cam4.4013 (PMC8267124; doi:10.1002/cam4.4013)
Supplement: Supplementary file 1 — Supplementary Material [file CAM4-10-4177-s001.docx]

**Supplemental material**

TITLE: Vitamin D supplementation for children with cancer: a systematic review and consensus recommendations.

**Table of contents**

Supplemental Table 1. Search strategies for the Pubmed, Embase and Cochrane databases. (pp. 2-4)

Supplemental Table 2. Critical appraisal of the observational studies using the QUIPS risk of bias tool. (p. 5)

Supplemental Table 3. Critical appraisal of the interventional studies using the Cochrane risk of bias tool. (p. 6)

Supplemental Table 4. GRADE assessment of the total body of observational and interventional studies. (pp. 7-8)

**Supplemental Table 1. Search strategies for the Pubmed, Embase and Cochrane databases.**

1) Pubmed

| Search 1:  Childhood cancer | Leukemia[MeSH] OR leukemi* [tiab] OR leukaemi* [tiab] OR “childhood ALL” [tiab] OR AML [tiab] OR lymphoma [MeSH] OR lymphom* [tiab] OR hodgkin* [tiab] OR non-hodgkin* [tiab] OR sarcoma [MeSH] OR sarcom* [tiab] OR “sarcoma, Ewing” [MeSH] OR Ewing* [tiab] OR osteosarcom* [tiab] OR “Wilms Tumor” [MeSH] OR wilms* [tiab] OR nephroblastom* [tiab] OR neuroblastoma [MeSH] OR neuroblastom* [tiab] OR rhabdomyosarcoma [MeSH] OR rhabdomyosarcom* [tiab] OR teratoma [MeSH] OR teratom* [tiab] OR “carcinoma, hepatocellular” [MeSH] OR hepatom* [tiab] OR hepatocellular* [tiab] OR hepatoblastoma [MeSH] OR hepatoblastom* [tiab] OR “neuroectodermal tumors, primitive” [MeSH] OR PNET* [tiab] OR medulloblastoma [MeSH] OR medulloblastom* [tiab] OR retinoblastoma [MeSH] OR retinoblastom* [tiab] OR meningioma [MeSH] OR “brain neoplasms” [MeSH] OR “central nervous system neoplasms” [MeSH] OR glioma [MeSH] OR brain tumor* [tiab] OR brain tumour* [tiab] OR “brain cancer” [tiab] OR brain neoplasm* [tiab] OR central nervous system neoplasm* [tiab] OR “central nervous system cancer” [tiab] OR central nervous system tumor* [tiab] OR central nervous system tumour* [tiab] OR intracranial tumor* [tiab] OR intracranial tumour* [tiab] OR “intracranial cancer” [tiab] OR intracranial neoplasm* [tiab] OR meningiom* [tiab] OR gliom*[tiab] OR “childhood oncology” [tiab] OR “pediatric oncology” [tiab] OR “paediatric oncology” [tiab] OR “childhood cancer” [tiab] OR “pediatric cancer” [tiab] OR “paediatric cancer” [tiab] OR childhood tumor* [tiab] OR pediatric tumor* [tiab] OR paediatric tumor* [tiab] OR childhood tumour* [tiab] OR pediatric tumour* [tiab] OR paediatric tumour* [tiab] OR childhood neoplasm* [tiab] OR pediatric neoplasm * [tiab] OR paediatric neoplasm* [tiab] OR childhood malignanc* [tiab] OR pediatric malignanc* [tiab] OR paediatric malignanc* [tiab] | |
| --- | --- | --- |
| Search 2:  Children | Pediatrics [MeSH] OR “young adult” [MeSH] OR child [MeSH] OR adolescent [MeSH] OR infan* [tiab] OR toddler* [tiab] OR minors [MeSH] OR minor* [tiab] OR boy* [tiab] OR girl* [tiab] OR kid* [tiab] OR child* [tiab] OR schoolchild* [tiab] OR adolescen* [tiab] OR juvenil* [tiab] OR youth* [tiab] OR teen* [tiab] OR pubescen* [tiab] OR pediatric* [tiab] OR paediatric* [tiab] | |
| Search 3:  Survivors | “Cancer survivors” [MeSH] OR survival [MeSH] OR surviv* [tiab] | |
| Search 4:  Late effects | “Long term adverse effects” [MeSH] OR “late effect” [tiab] OR “late effects” [tiab] OR “late side effect” [tiab] OR “late side effects” [tiab] OR “late adverse effect” [tiab] OR “late adverse effects” [tiab] OR “long term” [tiab] OR aftercare [tiab] OR “follow up” [tiab] | |
| Search 5:  Vitamin D | "Vitamin d"[MeSH] OR "vitamin d"[tiab] OR "calciferol"[tiab] OR "vitamin d3"[tiab] OR "vitamin d2"[tiab] OR cholecalciferol [tiab] OR calcitriol [tiab] OR calcefediol [tiab] OR 25 ohd* [tiab] OR 1,25(OH)2D [tiab] OR 24,25(OH)2D [tiab] OR marker* [tiab] OR biomarker* [tiab] OR laborator* [tiab] OR blood* [tiab] OR serum level* [tiab] | |
| Search 6:  Outcome | Osteoporosis [MeSH] OR osteoporos* [tiab] OR osteopeni* [tiab] OR "bone mineral density" [tiab] OR "bone density" [tiab] OR "bone loss" [tiab] OR "bone health" [tiab] OR "bone turnover" [tiab] OR "bone morbidity" [tiab] OR "bone morbidities" [tiab] OR "bone fragility" [tiab] OR "bone mass" [tiab] OR fracture* [tiab] OR "broken bone" [tiab] | |
| Combined: | 1 AND (2 OR 3 OR 4) AND 5 AND 6 | = 320 hits |

2) Embase

| Search 1:  Childhood cancer | 'Leukemia'/exp OR ‘lymphoma’/exp OR ‘nephroblastoma’/exp OR ‘sarcoma’/exp OR ‘Ewing sarcoma’/exp OR ‘meningioma’/exp OR ‘retinoblastoma’/exp OR ‘neuroblastoma’/exp OR ‘teratoma’/exp OR ‘liver cell carcinoma’/exp OR ‘hepatoblastoma’/exp OR ‘medullablastoma’/exp OR ‘glioma’/exp OR ‘central nervous system tumor’/exp OR ‘childhood cancer’/exp OR ‘childhood leukemia’/exp OR ‘acute lymphoblastic leukemia’/exp OR ‘acute myeloid leukemia’/exp OR ‘Hodgkin disease’/exp OR ‘neuroectoderm tumor’/exp OR leukemi*:ab,ti OR leukaemi*:ab,ti OR ‘childhood ALL’:ab,ti OR AML:ab,ti OR lymphom*:ab,ti OR hodgkin*:ab,ti OR non-hodgkin*:ab,ti OR sarcom*:ab,ti OR Ewing*:ab,ti OR osteosarcom*:ab,ti OR wilms*:ab,ti OR nephroblastom*:ab,ti OR neuroblastom*:ab,ti OR rhabdomyosarcom*:ab,ti OR teratom*:ab,ti OR hepatom*:ab,ti OR hepatocellular*:ab,ti OR hepatoblastom*:ab,ti OR PNET*:ab,ti OR medulloblastom*:ab,ti OR retinoblastom*:ab,ti OR ‘brain tumor*’:ab,ti OR ‘brain tumour*’:ab,ti OR ‘brain cancer’:ab,ti OR ‘brain neoplasm*’:ab,ti OR ‘central nervous system neoplasm*’:ab,ti OR ‘central nervous system cancer’:ab,ti OR ‘central nervous system tumor*’:ab,ti OR ‘central nervous system tumour*’:ab,ti OR ‘intracranial tumor*’:ab,ti OR ‘intracranial tumour*’:ab,ti OR ‘intracranial cancer’:ab,ti OR ‘intracranial neoplasm*’:ab,ti OR meningiom*:ab,ti OR gliom*:ab,ti OR ‘childhood oncology’:ab,ti OR ‘pediatric oncology’:ab,ti OR ‘paediatric oncology’:ab,ti OR ‘childhood cancer’:ab,ti OR ‘pediatric cancer’:ab,ti OR ‘paediatric cancer’:ab,ti OR ‘childhood tumor*’:ab,ti OR ‘pediatric tumor*’:ab,ti OR ‘paediatric tumor*’:ab,ti OR ‘childhood tumour*’:ab,ti OR ‘pediatric tumour*’:ab,ti OR ‘paediatric tumour*’:ab,ti OR ‘childhood neoplasm*’:ab,ti OR ‘pediatric neoplasm*’:ab,ti OR ‘paediatric neoplasm*’:ab,ti OR ‘childhood malignanc*’:ab,ti OR ‘pediatric malignanc*’:ab,ti OR ‘paediatric malignanc*’:ab,ti | |
| --- | --- | --- |
| Search 2:  Children | ‘Child’/exp OR ‘infant’/exp OR ‘adolescent’/exp OR ‘pediatrics’/exp OR infan*:ab,ti OR toddler*:ab,ti OR minor*:ab,ti OR boy*:ab,ti OR girl*:ab,ti OR kid*:ab,ti OR child*:ab,ti OR schoolchild*:ab,ti OR adolescen*:ab,ti OR juvenil*:ab,ti OR youth*:ab,ti OR teen*:ab,ti OR pubescen*:ab,ti OR pediatric*:ab,ti OR paediatric*:ab,ti | |
| Search 3:  Survivors | ‘Childhood cancer survivor’/exp OR ‘cancer survival’/exp OR ‘survival’/exp OR surviv*:ab,ti | |
| Search 4:  Late effects | ‘long term survival’/exp OR ‘late effect’:ab,ti OR ‘late effects’:ab,ti OR ‘late side effect’:ab,ti OR ‘late side effects’:ab,ti OR ‘late adverse effect’:ab,ti OR ‘late adverse effects’:ab,ti OR ‘long term’:ab,ti OR aftercare:ab,ti OR ‘follow up’:ab,ti | |
| Search 5:  Vitamin D | ‘Vitamin D’/exp OR ‘vitamin d’:ab,ti OR ‘calciferol’:ab,ti OR ‘vitamin d3’:ab,ti OR ‘vitamin d2’:ab,ti OR ‘cholecalciferol’:ab,ti OR ‘calcitriol’:ab,ti OR ‘calcefediol’:ab,ti OR ‘25 ohd*’:ab,ti OR 1,25OH2D:ab,ti OR 24,25OH2D:ab,ti OR marker*:ab,ti OR biomarker*:ab,ti OR laborator*:ab,ti OR blood*:ab,ti OR ‘serum level*’:ab,ti | |
| Search 6: Outcome | ‘Osteoporosis’/exp OR osteoporos*:ab,ti OR osteopeni*:ab,ti OR ‘bone mineral density’:ab,ti OR ‘bone density’:ab,ti OR ‘bone loss’:ab,ti OR ‘bone health’:ab,ti OR ‘bone turnover’:ab,ti OR ‘bone morbidity’:ab,ti OR ‘bone morbidities’:ab,ti OR ‘bone fragility’:ab,ti OR ‘bone mass’:ab,ti OR fracture*:ab,ti OR ‘broken bone’:ab,ti | |
| Combined: | 1 AND (2 OR 3 OR 4) AND 5 AND 6 | = 1219 hits |

3) Cochrane

| Search 1:  Childhood cancer | Leukemi*:ab,ti OR leukaemi*:ab,ti OR ‘childhood ALL’:ab,ti OR AML:ab,ti OR lymphom*:ab,ti OR hodgkin*:ab,ti OR non-hodgkin*:ab,ti OR sarcom*:ab,ti OR Ewing*:ab,ti OR osteosarcom*:ab,ti OR wilms*:ab,ti OR nephroblastom*:ab,ti OR neuroblastom*:ab,ti OR rhabdomyosarcom*:ab,ti OR teratom*:ab,ti OR hepatom*:ab,ti OR hepatocellular*:ab,ti OR hepatoblastom*:ab,ti OR PNET*:ab,ti OR medulloblastom*:ab,ti OR retinoblastom*:ab,ti OR ‘brain tumor*’:ab,ti OR ‘brain tumour*’:ab,ti OR ‘brain cancer’:ab,ti OR ‘brain neoplasm*’:ab,ti OR ‘central nervous system neoplasm*’:ab,ti OR ‘central nervous system cancer’:ab,ti OR ‘central nervous system tumor*’:ab,ti OR ‘central nervous system tumour*’:ab,ti OR ‘intracranial tumor*’:ab,ti OR ‘intracranial tumour*’:ab,ti OR ‘intracranial cancer’:ab,ti OR ‘intracranial neoplasm*’:ab,ti OR meningiom*:ab,ti OR gliom*:ab,ti OR ‘childhood oncology’:ab,ti OR ‘pediatric oncology’:ab,ti OR ‘paediatric oncology’:ab,ti OR ‘childhood cancer’:ab,ti OR ‘pediatric cancer’:ab,ti OR ‘paediatric cancer’:ab,ti OR ‘childhood tumor*’:ab,ti OR ‘pediatric tumor*’:ab,ti OR ‘paediatric tumor*’:ab,ti OR ‘childhood tumour*’:ab,ti OR ‘pediatric tumour*’:ab,ti OR ‘paediatric tumour*’:ab,ti OR ‘childhood neoplasm*’:ab,ti OR ‘pediatric neoplasm*’:ab,ti OR ‘paediatric neoplasm*’:ab,ti OR ‘childhood malignanc*’:ab,ti OR ‘pediatric malignanc*’:ab,ti OR ‘paediatric malignanc*’:ab,ti | |
| --- | --- | --- |
| Search 2:  Children | Infan*:ab,ti OR toddler*:ab,ti OR minor*:ab,ti OR boy*:ab,ti OR girl*:ab,ti OR kid*:ab,ti OR child*:ab,ti OR schoolchild*:ab,ti OR adolescen*:ab,ti OR juvenil*:ab,ti OR youth*:ab,ti OR teen*:ab,ti OR pubescen*:ab,ti OR pediatric*:ab,ti OR paediatric*:ab,ti | |
| Search 3:  Survivors | Surviv*:ab,ti | |
| Search 4:  Late effects | ‘Late effect’:ab,ti OR ‘late effects’:ab,ti OR ‘late side effect’:ab,ti OR ‘late side effects’:ab,ti OR ‘late adverse effect’:ab,ti OR ‘late adverse effects’:ab,ti OR ‘long term’:ab,ti OR aftercare:ab,ti OR ‘follow up’:ab,ti | |
| Search 5:  Vitamin D | ‘Vitamin d’:ab,ti OR ‘calciferol’:ab,ti OR ‘vitamin d3’:ab,ti OR ‘vitamin d2’:ab,ti OR ‘cholecalciferol’:ab,ti OR ‘calcitriol’:ab,ti OR ‘calcefediol’:ab,ti OR ‘25 ohd*’:ab,ti OR 1,25OH2D:ab,ti OR 24,25OH2D:ab,ti OR marker*:ab,ti OR biomarker*:ab,ti OR laborator*:ab,ti OR blood*:ab,ti OR ‘serum level*’:ab,ti | |
| Search 6: Outcome | Osteoporos*:ab,ti OR osteopeni*:ab,ti OR ‘bone mineral density’:ab,ti OR ‘bone density’:ab,ti OR ‘bone loss’:ab,ti OR ‘bone health’:ab,ti OR ‘bone turnover’:ab,ti OR ‘bone morbidity’:ab,ti OR ‘bone fragility’:ab,ti OR ‘bone mass’:ab,ti OR ‘bone morbidities’:ab,ti OR fracture*:ab,ti OR ‘broken bone’:ab,ti | |
| Combined**:** | 1 AND (2 OR 3 OR 4) AND 5 AND 6 | **= 109** hits |

**Supplemental Table 2. Critical appraisal of the observational studies using the QUIPS risk of bias tool.**

*Validity*

|  | Study participation | Study attrition | PF measurement | Outcome measurement | Study confounding | Statistical analysis and reporting |  |  |
| --- | --- | --- | --- | --- | --- | --- | --- | --- |
| Bilariki 2010 | 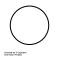 | 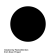 | 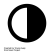 | 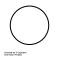 | 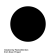 | 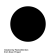 |  |  |
| Boot 1999 | 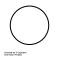 | 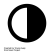 | 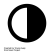 | 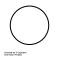 | 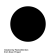 | 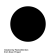 |  |  |
| Bordbar 2016 | 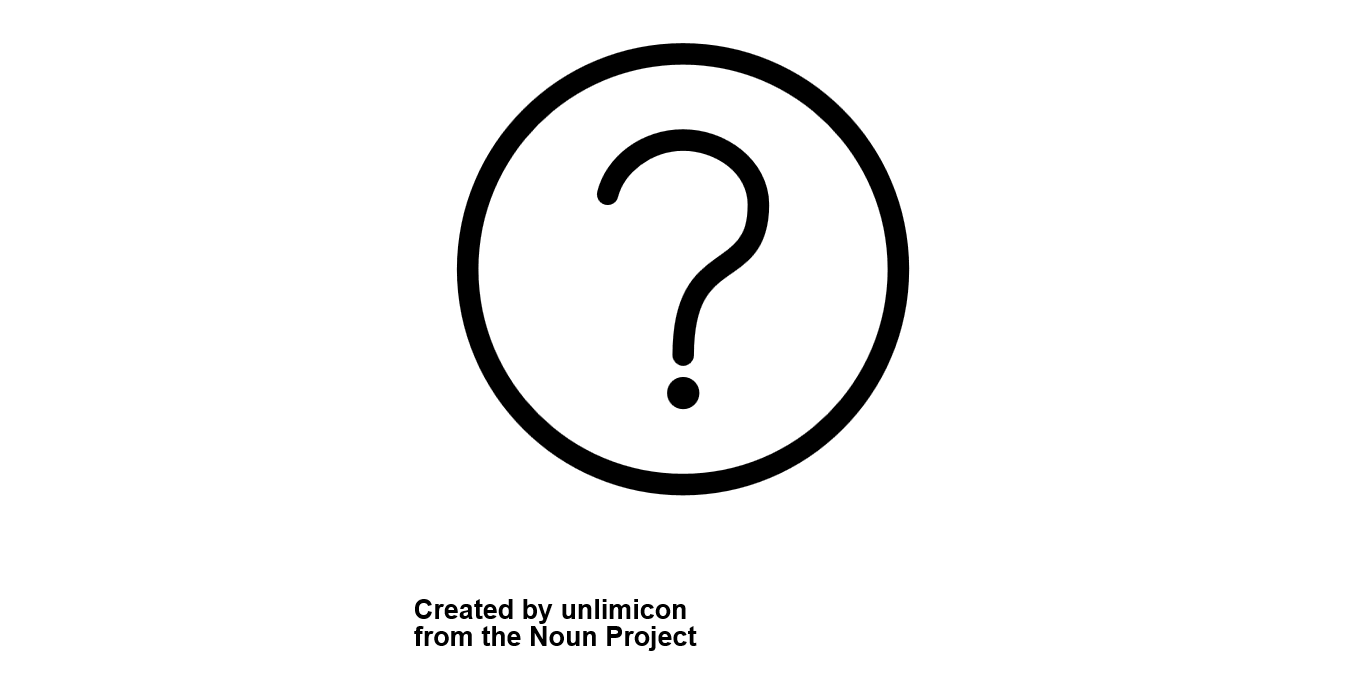 | 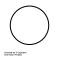 | 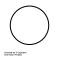 | 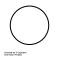 | 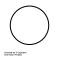 | 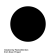 |  |  |
| Choi 2017 | 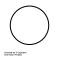 | 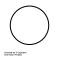 | 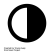 | 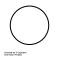 | 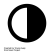 | 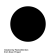 |  |  |
| El-Ziny 2005 | 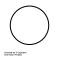 | 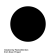 | 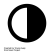 | 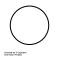 | 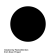 | 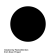 |  |  |
| El-Ziny 2007 | 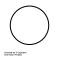 | 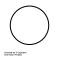 | 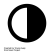 | 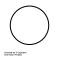 | 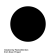 | 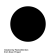 |  |  |
| Esbenshade 2014 | 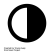 | 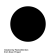 | 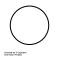 | 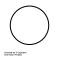 | 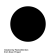 | 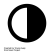 |  |  |
| Gunes 2010 | 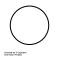 | 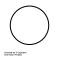 | 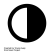 | 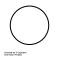 | 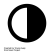 | 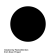 |  |  |
| Halton 1995 | 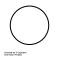 | 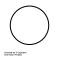 | 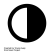 | 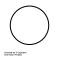 | 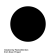 | 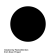 |  |  |
| Henderson 1998 | 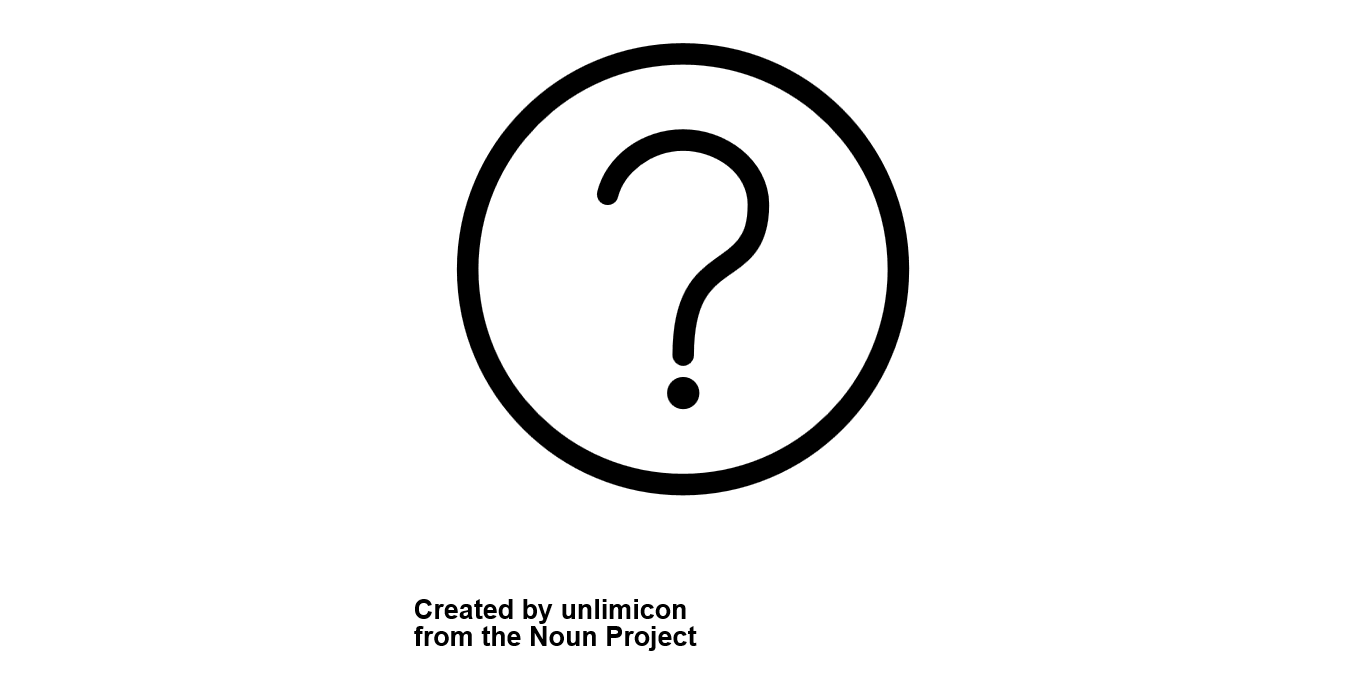 | 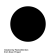 | 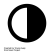 | 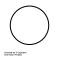 | 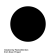 | 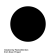 |  |  |
| Jain 2017 | 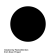 | 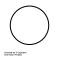 | 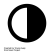 | 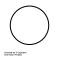 | 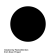 | 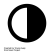 |  |  |
| Kadan-Lottick 2001 | 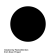 | 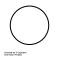 | 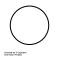 | 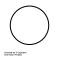 | 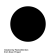 | 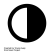 |  |  |
| Kelly 2009 | 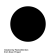 | 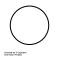 | 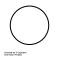 | 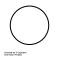 | 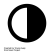 | 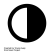 |  |  |
| Marinovic 2005 | 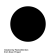 | 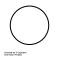 | 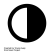 | 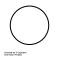 | 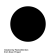 | 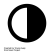 |  |  |
| Mostoufi-Moab 2012 | 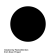 | 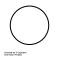 | 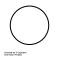 | 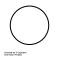 | 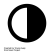 | 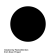 |  |  |
| Saki 2018 | 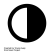 | 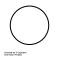 | 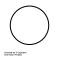 | 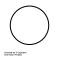 | 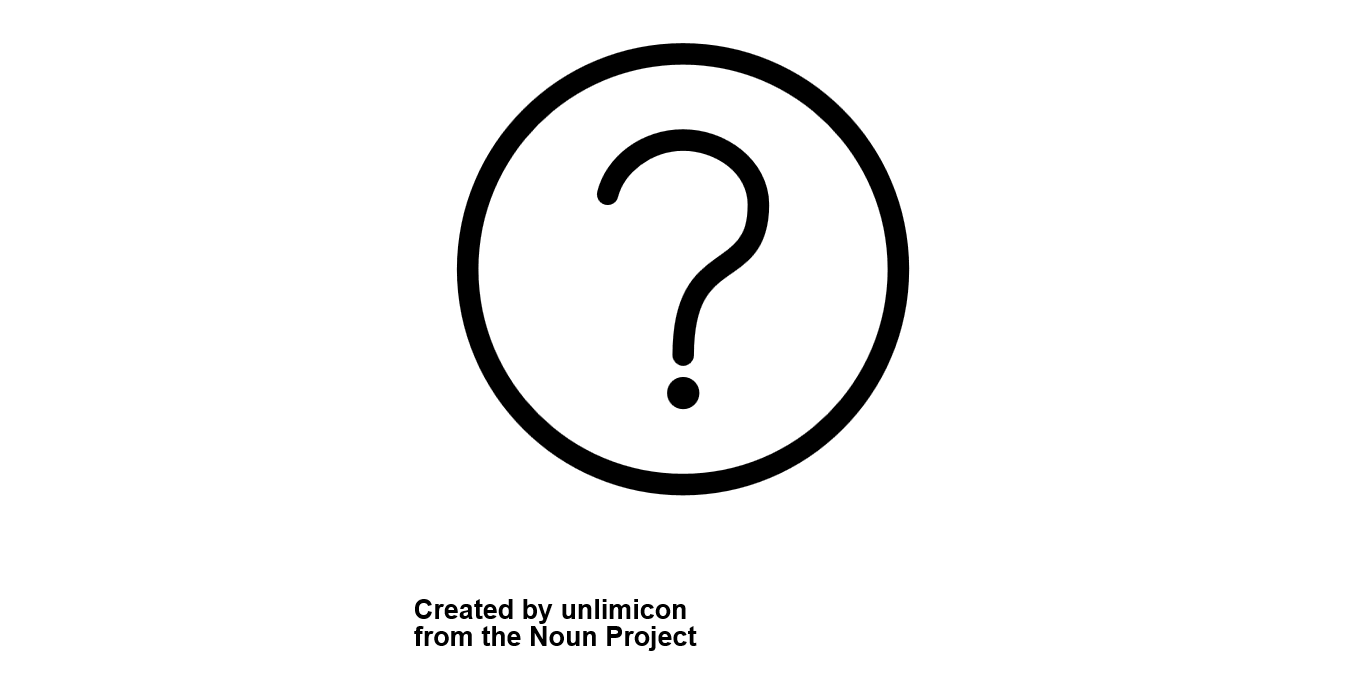 | 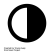 |  |  |

| 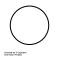 | Low risk of bias |
| --- | --- |
| 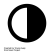 | Moderate risk of bias |
| 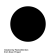 | High risk of bias |
| 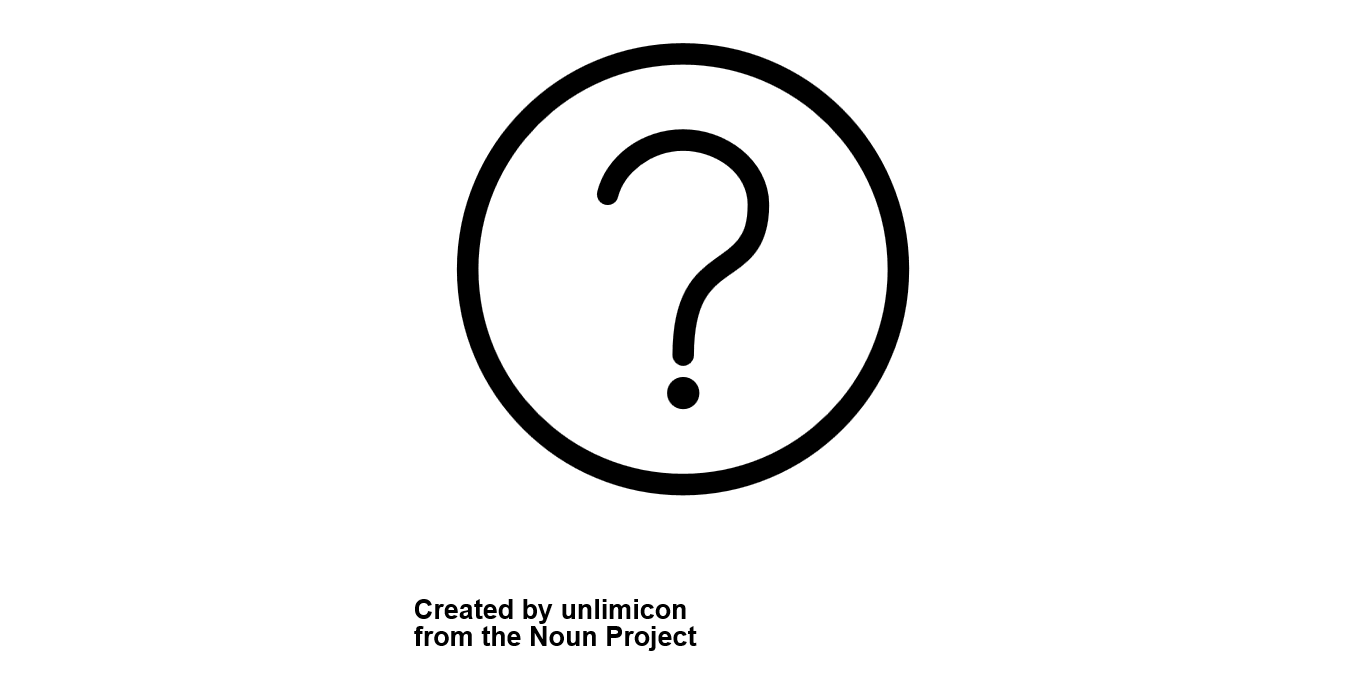 | Risk of bias unclear |

PF=prognostic factor

**Supplemental Table 3. Critical appraisal of the interventional studies using the Cochrane risk of bias tool.**

*Validity*

|  | Random sequence generation | Allocation concealment | Blinding of participants and personnel | Blinding of outcome assessment | Incomplete outcome data | Selective reporting | Other bias |  |
| --- | --- | --- | --- | --- | --- | --- | --- | --- |
| Demirsoy 2017 |  |  |  |  |  |  |  |  |
| Díaz 2008 |  |  |  |  |  |  |  |  |
| Orgel 2017 |  |  |  |  |  |  |  |  |

|  | Low risk of bias |
| --- | --- |
|  | High risk of bias |
|  | Risk of bias unclear |

**Supplemental Table 4. GRADE assessment of the total body of observational and interventional studies.**

| **Quality assessment** | | | | | | | | | | | | | |
| --- | --- | --- | --- | --- | --- | --- | --- | --- | --- | --- | --- | --- | --- |
| **Study design** | **Study limitations** | | **Inconsistency** | **Indirectness** | **Imprecision** | **Publication bias** | | **Effect size** | | **Dose-response** | | | **Plausible confounding** |
| Observational studies  *What is the risk of lower BMD Z-scores for lower serum 25OHD levels in children with cancer up to five years after cancer therapy?* | | | | | | | | | | | | | |
| Longitudinal and cross-sectional cohort studies^1^  (+4) | Very serious^2^  (-2) | | Serious^3^  (-1) | Not serious | Serious^4^  (-1) | Unlikely | | No large effect size | | Dose-response effect not assessed | | | No plausible confounding |
| **Overall quality of the evidence:** ⊕⊖⊖⊖ VERY LOW  **Conclusion:** There is conflicting evidence for the association between lower serum 25OHD levels and lower BMD Z-scores in children with cancer up to five years after cancer therapy.  **Number of studies and participants:** 16 studies, 873 participants | | | | | | | | | | | | | |
| Observational studies  *What is the risk of fractures for lower serum 25OHD levels in children with cancer up to five years after cancer therapy?* | | | | | | | | | | | | | |
| Cross-sectional cohort studies^5^  (+2) | | Very serious^2^  (-2) | Not serious | Not serious | Serious^4^  (-1) | | Unlikely | | No large effect size | | Dose-response effect not assessed | No plausible confounding | |
| **Overall quality of the evidence:** ⊕⊖⊖⊖ VERY LOW  **Conclusion:** No increased risk of fractures for lower serum 25OHD levels in children with cancer up to five years after cancer therapy.  **Number of studies and participants:** 2 studies, 89 participants | | | | | | | | | | | | | |
| Interventional studies  *What is the effect of vitamin D supplementation on BMD and fractures in children with cancer up to five years after cancer therapy?* | | | | | | | | | | | | | |
| Two RCTs, one quasi-experimental study^6^  (+2) | | Very  Serious^7^  (-2) | Not serious | Serious^8^  (-1) | Serious^4^  (-1) | | Unlikely | No large effect size | | | Dose-response effect not assessed | | No plausible confounding |
| **Overall quality of the evidence:** ⊕⊖⊖⊖ VERY LOW  **Conclusion:** No significant effect of vitamin D supplementation on BMD and fractures in children with ALL up to five years after cancer therapy compared to controls.  **Number of studies and participants:** 3 studies, 61 participants, 77 controls | | | | | | | | | | | | | |

BMD=bone mineral density; RCT=randomized controlled trial

^1^Initial score +4 assigned, as evidence from these observational study designs is the best available for prognostic questions

^2^Downgraded for risk of bias, as the QUIPS tool showed significant risk of bias for study participation, prognostic factor measurement, study confounding and statistical analysis and reporting

^3^Downgraded for inconsistency, as one study reported a significant association between lower 25OHD levels and lower BMD

^4^Downgraded for imprecision, as the sample size of most studies was very small

^5^Initial score +2 assigned, as the studies were longitudinal for BMD but cross-sectional for fractures: the association between 25OHD levels and a *history* of fractures was assessed

^6^Initial score +2 assigned, as the majority of the studies was no RCT

^7^Downgraded for risk of bias, as the Cochrane Risk of Bias tool showed significant risk of bias for random sequence generation, allocation concealment, incomplete outcome data, and other bias

^8^Downgraded for indirectness, as all interventional studies were performed in children with acute lymphoblastic leukemia, and therefore the results might not be generalizable to children with other cancer diagnoses
